# Getting Ahead of St. Paul

**Published:** 1890-10

**Authors:** 


GETTING AHEAD OF ST. PAUL.—Dr. J. N. Crouse, of Chicago, is a multifarious-officed gentleman. He is president of the Dental Protective Association, chairman of the executive committee, chairman of the committee on arrangements, and hustler in general for all sorts of miscellaneous business. He is an exceedingly shrewd and smooth tempered man,one of that sort that is never ruffled, but always so dead in earnest that most folks think it but right to give him his own way without dispute, those who don't generally get the worst of it, and that is precisely what happened to the Chicago, Milwaukee & St. Paul railway on the day when the members of the convention arrived. Dr. Crouse, in his capacity as chairman of the executive committee, had to make the railroad arrangements. When he arrived at the Milwaukee depot in Kansas City, it happened that, through some misunderstanding or failure of other roads to make connections, there were only thirty or forty dentists to board the special train that had been ordered, while the railroad officials had expected at least a hundred. It was useless to represent that there were more than a hundred extra men on the way who would take the regular trains to Excelsior Springs. The officials refused point blank, and with what Dr. Crouse thought "most damnable iteration,"to take the trains out unless $50 was paid in addition to the fares already represented by tickets. Finally Dr. Crouse consented, the money was paid over, a receipt taken and the special dentists' train started. Just after crossing the river Dr. Crouse noticed the conductor collecting fares from people who had boarded the train at way stations. This was his opportunity. A sharp pull at the bell brought the train to a halt; it also brought the conductor to remonstrate. To that official's surprise he was taken in charge by the hustling doctor, who informed him that for the time being he must consider himself in the employ of the American Dental Association, producing the receipt for $50 in support of his statement. The doctor then compelled the conductor to return all fares collected and to remove the passengers that had paid them from the train. These unfortunate wights were dumped into a corn field and the doctors' train moved triumphantly on to Excelsior Springs.

